# TRPV2 Mediates Adrenomedullin Stimulation of Prostate and Urothelial Cancer Cell Adhesion, Migration and Invasion

**DOI:** 10.1371/journal.pone.0064885

**Published:** 2013-05-31

**Authors:** Agathe Oulidi, Alexandre Bokhobza, Dimitra Gkika, Fabien Vanden Abeele, V’yacheslav Lehen’kyi, L’Houcine Ouafik, Brigitte Mauroy, Natalia Prevarskaya

**Affiliations:** 1 INSERM U1003, Equipe Labellisée par la Ligue Nationale contre le Cancer, Villeneuve d’Ascq, France; 2 Laboratory of Excellence, Ion Channels Science and Therapeutics, Universite des Sciences et Technologies de Lille (USTL), Villeneuve d’Ascq, France; 3 Inserm UMR 911-CRO2, Faculté de Médecine Timone, Marseille, France; University of Kentucky College of Medicine, United States of America

## Abstract

Adrenomedullin (AM) is a 52-amino acid peptide initially isolated from human pheochromocytoma. AM is expressed in a variety of malignant tissues and cancer cell lines and was shown to be a mitogenic factor capable of stimulating growth of several cancer cell types. In addition, AM is a survival factor for certain cancer cells. Some data suggest that AM might be involved in the progression cancer metastasis via angiogenesis and cell migration and invasion control. The Transient Receptor Potential channel TRPV2 is known to promote in prostate cancer cell migration and invasive phenotype and is correlated with the stage and grade of bladder cancer. In this work we show that AM induces prostate and urothelial cancer cell migration and invasion through TRPV2 translocation to plasma membrane and the subsequent increase in resting calcium level.

## Introduction

Adrenomedullin (AM) is a 52 amino acid peptide originally isolated from a human pheochromocytoma [1] that bears multifactorial regulatory properties ranging from inducing vasodilatation to modulating cellular growth [2]. The functions of AM are mediated through specific receptors comprising calcitonin receptor-like receptor (CLR) and a receptor activity-modifying protein (RAMP); when co-expressed with RAMP2 or RAMP3, CLR functions as a specific AM receptor [3]. AM and its receptors are highly expressed in various cancer cell lines and in cancers of the pancreas, lung, kidney, breast, ovary and prostate. A number of studies have implicated AM (secreted endogenously or exogenously administered) in tumor growth, progression and metastasis via effects on angiogenesis, cell proliferation, apoptosis and migration [4]–[6]. However, molecular mechanisms underlying these effects of AM on cancer cell growth and metastasis remain contradictory and poorly understood.

Cell migration plays a pivotal role in cancer invasion and metastasis. Many of the components of cellular migration machinery are regulated by the intracellular calcium (Ca^2+^) concentration [7]. An essential part of the intracellular Ca^2+^ signal is generated by the transmembrane influx of extracellular Ca^2+^ mainly occurring through cationic channels with distinctive Ca^2+^ selectivity. In non-excitable cells, Ca^2+^ entry is provided by ion channels that are activated by various chemical and physical stimuli. Some of these Ca^2+^ entry channels are members of the “transient receptor potential” (TRP) family of cationic channels [8]. TRP channels have been reported to be involved in carcinogenesis [9]–[11], and among them TRPV2 channel, has been shown to be specifically implicated in the progression of prostate and bladder cancers to more aggressive phenotype. Indeed, recent studies from our laboratory have shown show that TRPV2 is expressed in the more aggressive prostate cancer cells and stimulates the migration and invasive phenotype of these cells [12], [13]. Interestingly, TRPV2 expression in bladder is also shown to correlate with the grade and stage of cancer [14] but nothing is known about its potential role in bladder cancer cell migration/invasion.

In the present work we investigated the involvement of TRPV2 in the effect of AM on the multistep process of invasion in two highly invasive cell lines: the PCa (Prostate Cancer) cells PC-3 and the UC (Urothelial Carcinoma) cells T24/83. Our data indicate that AM can increase adhesion, migration and invasion through Focal Adhesion Kinase (FAK) and integrin β1 activation, and the stimulation of TRPV2 translocation to the plasma membrane. Thus, our results provide new insights into the role of AM and TRPV2 in prostate and bladder malignancies.

## Materials and Methods

### Cell Culture

The human PCa cell line PC-3 was obtained from the ATCC and maintained in culture in RPMI 1640 (Life Technologies) supplemented with 10% FCS and 5 mM L-glutamine (Sigma). The urothelial cancer cell line T24/83 was obtained from the ECACC and maintained in culture in McCoy’s 5A Glutamax® (Life technologies) supplemented with 10% FCS.

### Reverse Transcription-PCR

Total mRNA was isolated from cells as previously described [12]. DNA amplification conditions included an initial denaturation step of 7 min at 95°C; 35 cycles of 30 sec at 95°C, 30 sec at 60°C, and 30 sec at 72°C; and finally 7 min at 72°C. Primers sequences and sizes of fragments was for RAMP2: forward 5′-CTCAGCCTCTTCCCACCAC-3′, reverse 5′-TTCCAGCAAAATTGGACAGC-3′, 84 bp; for RAMP3 5′-ATCTCGGTGCAGTTGGTGA-3′ and 5′- AAGGTGGACGTCTGGAAGTG-3′, 77 bp; for CLR 5′-CATGGACAAATTATACCCAGTGT-3′ and 5′-TCCAATTATGGTCAGGTAAAACAA3’, 86 bp; for Actin 5′-CAGAGCAAGAGAGGCATCCT-3′ and 5′-GTTGAAGGTCTCAAACATGATC-3′, 209 bp.

### Small Interfering RNA Transfection

PC-3 and T24/83 cells were transfected with 50 nM small interfering RNA (siRNA) against TRPV2 (siTRPV2 sequence: 5′-UAAGAGUCAACCUCAACUAdT-3′, synthesized by Eurogentec) using HiPerFect transfection reagent (Qiagen) following the manufacturer’s instruction.

### Cell Adhesion Assay

Cells were harvested and seeded at 3*10^4^ and 1.5*10^4^ cells/well for PC3 and T24/83 respectively in basal medium supplemented or not with AM (200 nM) on fibronectin (10 µg/ml) pre-coated 96-well plates. After 45min of incubation at 37°C, adhered cells were fixed 15 minutes in methanol bath and stained with Hoechst (5 mg/ml in PBS), photos were taken on a Leica DMIRE2 microscope (×5) and cells counted using the NIH image analysis software (ImageJ).

### Cell Migration and Invasion Assay

Cell migration and invasion was determined by transwell assay. Briefly, Cells were seeded on top of Transwell cell culture inserts with 8 µm pore size (Falcon) at a density of 60,000 per well for PC-3 and 30,000 for T24/83 (24-well format) in serum-free culture medium. After 1 h the molecule of interest was added on both sides of the Transwell filter. For the invasion assay, the upper compartment was coated with 50 µg Matrigel (BD Biosciences) to form a matrix barrier. The lower compartment was filled with medium containing 10% FCS as chemoattractant. After 8 h for the migration assay and 24 h for invasion, non-migratory cells were removed from the top filter by scraping, whereas cells that had migrated through the filter pores to the lower face of the inserts were fixed in 4% paraformaldehyde in PBS and stained with Hoechst (5 mg/ml in PBS) and counted using a Leica DMIRB microscope (×200).

### Ca^2+^ Measurements using Fura-2 AM

Prior to fluorescence measurements, the cells were trypsinized and plated onto glass slips. The medium was replaced every 48 h. Cells were used 3 days after trypsinization. The culture medium was replaced by a HBSS solution containing 142 mmol/L NaCl, 5.6 mmol/L KCl, 1 mmol/L MgCl2, 2 mmol/L CaCl2, 0.34 mmol/L Na2HPO4, 0.44 mmol/L KH2PO4, 10 mmol/L HEPES, and 5.6 mmol/L glucose. The osmolarity and pH of this solution were adjusted to 310 mOsm/L and 7.4, respectively. Dye loading was achieved by transferring the cells into a standard HBSS solution containing 1 mmol/L Fura-2 acetoxymethyl ester (Calbiochem) loaded (45 min) for 40 min at 37°C. Subsequently, cells were washed three times with the same dye-free solution. The coverslip was then transferred onto a perfusion chamber on a Olympus IX70 microscope equipped for fluorescence. Fluorescence was alternatively excited at 340 and 380 nm with a monochromator and was captured after filtration through a long-pass filter (510 nm) by a MicroMax 5 MHz CCD camera (Princeton Instruments, Evry, France). Acquisition and analysis was performed with the Metafluor 7.7.4.0 software (Universal Imaging Corp., West Chester, PA). The intracellular calcium concentration was derived from the ratio of the fluorescence intensities for each of the excitation wavelengths (F340/F380) and from the equation of Grynkiewicz. The cells were continuously perfused with HBSS solution via a whole-chamber perfusion system and chemicals were added via the perfusion system. The flow rate of the whole chamber perfusion system was set to 1 ml/min and the chamber volume was 500 µl. All recordings were made at 37°C.

### Biotinylation and Western-blotting

The experiments were carried out as described previously [15]. Antibodies used were rabbit polyclonal anti-VRL-1 (for TRPV2, 1/200, Santa Cruz), anti-FAK (1/500, abcam), anti-FAK phosphor Y397 (1/1000, abcam), anti-integrin beta 1 phospho T788+T789 (1/1000, abcam), and anti-integrin beta 1 (1/200, Santa Cruz), and mouse monoclonal anti-beta-actin (1/2000, Sigma-Aldrich).

### Data Analysis

Results were expressed as mean ± SE. Plots were produced using Excel. Each experiment was repeated at least 3 times. n indicates the number of cells per experiment. N indicates the number of experiments performed. The Turkey-Kramer test was used for statistical comparison among means and differences, and P<0.05 was considered significant.

## Results

### Adrenomedullin Increases PC-3 and T24/83 Cell Adhesion, Migration and Invasion

**Figure 1:**
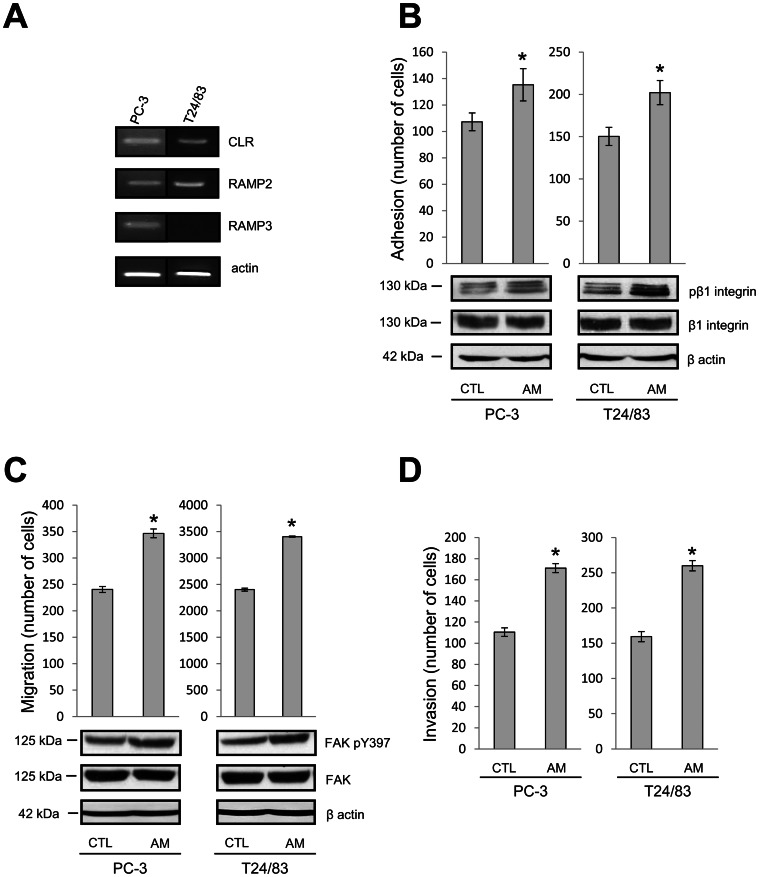
Adrenomedullin increases PC-3 and T24/83 cell adhesion, migration and invasion. (A) RT-PCR experiment showing RAMP2, RAMP3 and CLR expression in PC-3 and T24/83 cells. (B) PC-3 (left panel) and T24/83 (right panel) cell adhesion was examined by seeding 3*10^4^ and 1.5*10^4^ cells respectively per well in 96-well plates pre-coated with fibronectin, and incubated for 45 min with or without AM (200 nM) (N = 3, *P<0.05 compared with control cells). β1 integrin phosphorylation was studied by western-blotting on total proteins extracted from PC-3 and T24/83 cells seeded on fibronectin coated plates and treated with or without AM. (C) PC-3 and T24/83 cell migration was studied by Transwell assay after 8 h of treatment (N = 3, *P<0.05 compared with control cells). FAK phosphorylation was studied by western-blotting on total proteins extracted from PC-3 and T24/83 cells treated with or without AM. (D) For invasion assay, transwell membrane was pre-coated with 50 µg Matrigel, and PC-3 and T24/83 cells were let to invade for 24 h (N = 3, *P<0.05 compared with control cells).

We first checked by RT-PCR the expression of AM receptors in the prostate and urothelial cancer cell lines PC3 and T24/83. As shown in figure 1A, PC-3 expresses the two AM receptors, CLR/RAMP2 and CLR/RAMP3, whereas T24/83 expresses only CLR/RAMP3.

Then we investigated the cell adhesion to a major component of the basal membrane, the fibronectin. Addition of 200 nM AM increased both PC-3 and T24/83 cells by 26% and 34% respectively (Fig. 1B). One of the major molecular players modifying cell-substrate adhesion is the integrin superfamily of receptors, which consist of heterodimers of α- and β- subunits recognizing different extracellular matrix (ECM) proteins. Interestingly, β1-integrin is the most abundant subunit expressed in PCa cells and is capable of forming heterodimers binding to fibronectin [16], while increased β1-integrin expression correlates with more invasive and metastatic bladder cancer [17]. Activation of β1-integrin leads to its phosphorylation, which can be examined by western blotting. We therefore tested whether AM could activate β1-integrin by studying its phosphorylation on Thr-788-789 with a phospho-specific antibody. We observed that AM treatment increased significantly the level of the phosphorylated β1-integrin without affecting the expression of the total protein form (Fig. 1B, lower panel).

Modification of adhesion might promote migration and invasion: two important malignancy-associated phenotypes. So, we examined the effect of AM treatment on these phenotypes by using transwell assays. Addition of 200 nM AM increased the migration of PC-3 by 45% and T24/83 cells by 40% (Fig. 1C). Several studies showed that upon engagement with components of the ECM, integrins clustered leading to the activation of focal adhesion kinase (FAK) by autophosphorylation at Tyr397. The activation of FAK controls cell shape and motility [18]. We studied thus the activity of FAK by western-blotting with a specific antibody against phospho-Tyr397 FAK. As shown in Fig. 1C (lower panel), the phosphorylated FAK has been significantly increased by the AM treatment total FAK while the total FAK expression remained unchanged.

In addition, we studied the effect of AM on the invasion potential of the two cell lines by transwell assay for which the transwell filter was coated with Matrigel. AM treatment increased the ability of cell invasion through the Matrigel-coated membrane by 54% in PC-3 cells and by 61% in T24/83 cells (Fig. 1D).

### TRPV2 Mediates Adrenomedullin Promotion of Migration and Invasion on PC-3 and T24/83 Cells

**Figure 2:**
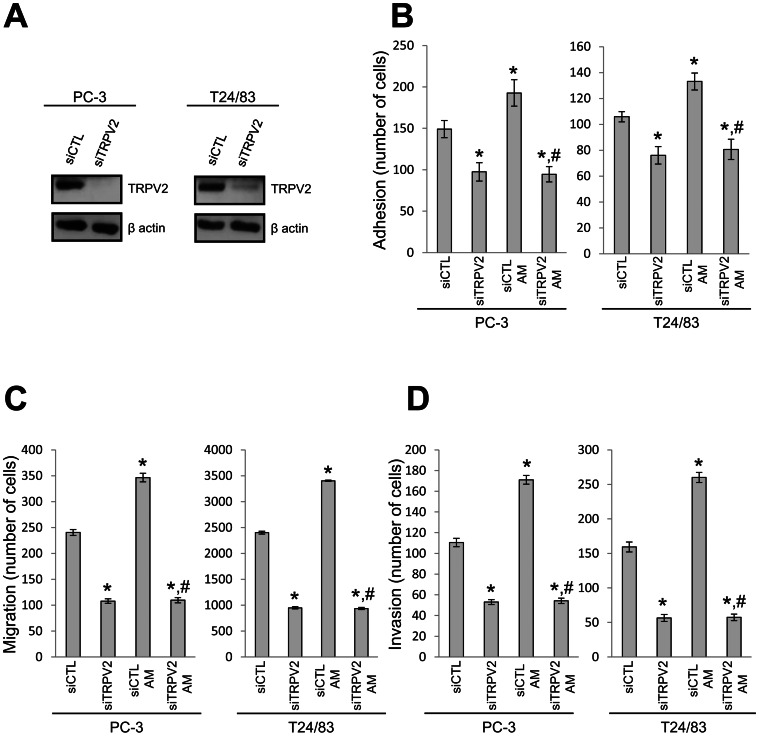
Adrenomedullin effect is mediated by TRPV2. (A) Western-blotting analysis of TRPV2 protein level in PC-3 and T24/83 cells treated with either siCTL or siTRPV2 (50 nM, 48 h). Effect of TRPV2 silencing (siTRPV2, 50 nM, 48 h) (B) on PC-3 and T24/83 cell adhesion to fibroncectin incubated or not with AM (200 nM, 45 min) (N = 3, *P<0.05 compared with control cells; ^#^P<0.05 compared with control cells treated with AM); (C) on PC-3 and T24/83 cell migration examined by transwell assay after 8 h incubation with or without AM (N = 3 *, P<0.05 compared with control cells; ^#^P<0.05 compared with control cells treated with AM); (D) on PC-3 and T24/83 cell invasion through matrigel (AM 200 nM, 24 h) (N = 3. *, P<0.05 compared with control cells; ^#^P<0.05 compared with control cells treated with AM).

Since, we have previously shown that TRPV2 was involved in PCa cell migration and invasiveness [12], [13] and since this channel was also implicated in bladder carcinogenesis [14], we tested whether TRPV2 is implicated in the increase of cell migration and invasion by AM. We first checked TRPV2 expression in the two cell lines and its downrerulation by siRNA. Western-blot analysis with TRPV2-specific antibody has shown that TRPV2 protein is expressed in PC3 and T24/83 cells and that protein expression can be effectively inhibited by cells treatment with siRNA-TRPV2 (50 nM, 48 h, Fig. 2A). Silencing of TRPV2 with siRNA-TRPV2 not only decreased adhesion of PC-3 and T24/83 cells by 35% and 29% respectively, but also abolished stimulatory effects on adhesion by AM treatment (200 nM). AM addition to the cells treated with control siRNA was able to enhance their adhesion to fibronectin by 29% for PC-3 and 25% for T24/83 cells (Fig. 2B).

We next investigated the effect of the knock down of TRPV2 on cell migration by transwell assay. Downregulation of TRPV2 expression decreased not only PC-3 cell migration, but also T24/83, by 55% and 60% respectively. As was shown for the adhesion, AM treatment on cells treated with siRNA-TRPV2 was no longer able to promote migration (Fig. 2C).

Finally, we examined the invasion of PC-3 and T24/83 cells through the Matrigel-coated membrane and as observed for the adhesion and migration, siRNA-TRPV2 induced a decrease of both PC-3 and T24/83 invasion, by 52% and 67%. Addition of AM could not increase the invasion of PC-3 and T24/83 cells treated by siTRPV2 (Fig. 2D).

### AM Induces TRPV2 Translocation at the Plasma Membrane via a PI3K Pathway

**Figure 3:**
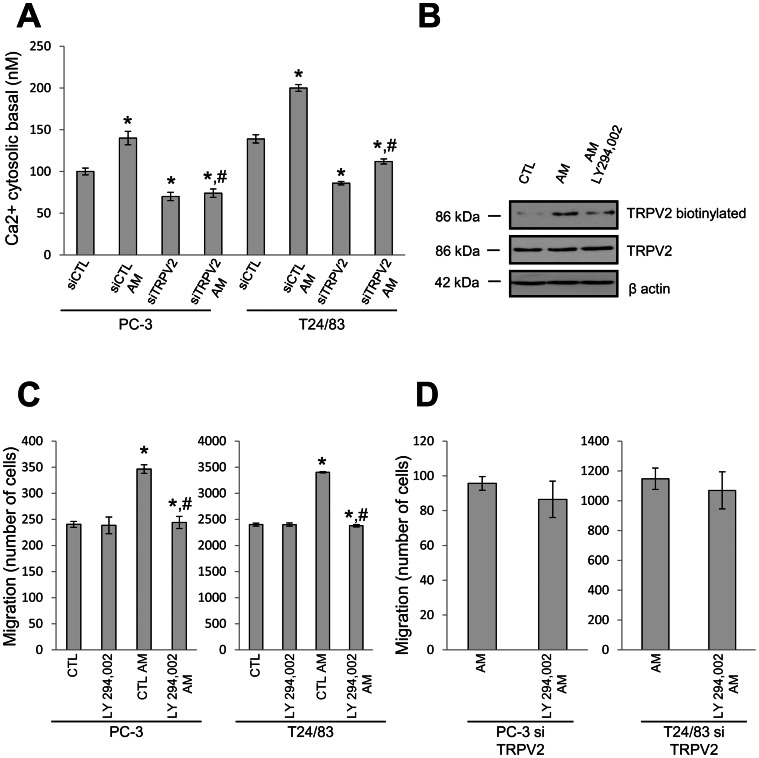
Adrenomedullin induces TRPV2 translocation to plasma membrane. (A) The effect of AM (200 nM, 45 min) and TRPV2 silencing (siTRPV2, 50 nM, 48 h) on basal cytosolic calcium of PC-3 and T24-83 cells was studied by calcium imaging. (n = 120 cells, N = 4, *, P<0.05 compared with control cells; ^#^P<0.05 compared with control cells treated with AM). (B) TRPV2 presence at the plasma membrane was examined by biotinylation on T24/83 cells control or either treated with AM (200 nM, 45 min) or AM and PI3K inhibitor LY294.002 (10 µM, added 5 min before AM). (C) Effect of LY294.002 on PC-3 and T24/83 cell migration examined by transwell assay after 8 h incubation with or without AM (N = 3. *, P<0.05 compared with control cells; ^#^P<0.05 compared with control cells treated with AM). (D) Effect of LY294.002 on AM-induced migration of PC-3 and T24/83 TRPV2-silenced cells examined by transwell assay after 8 h incubation with or without AM (N = 3. *, P<0.05 compared with control cells; ^#^P<0.05 compared with control cells treated with AM).

In view of TRPV2 involvement in AM effect on cell migration, we next examined by calcium imaging if AM could activate TRPV2. Acute application of AM to PC-3 and T24/83 did not cause elevation of intracellular Ca^2+^ concentration ([Ca^2+^]_i_) on a short time-scale (i.e., within minutes), as one would expect from enhanced TRPV2-mediated Ca^2+^ entry (data not shown). However, prolonged 45 min long treatment of the two cell lines with AM induced an increase of the basal [Ca^2+^]i level (PC-3: from control value of 100 nM to 140 nM in the presence of AM;T24/83∶139 nM to 200 nM; Fig. 3A). The silencing of TRPV2 by siRNA (50 nM, 48 h) decreased the basal [Ca^2+^]_i_ (PC-3: to 70 nM; T24/83: to 86 nM) suggesting that steady-state TRPV2-mediated Ca^2+^ influx contributes to the resting cytosolic Ca^2+^ concentration. Moreover, TRPV2 silencing also prevented the increase of basal [Ca^2+^]_I_ in response to the AM treatment (Fig. 3A), consistent with the notion that prolonged incubation of PC-3 and T24/83 cells causes indirect activation of TRPV2 by AM via signaling pathway that requires some time to produce its effects.

It is known that AM can activate PI3K [19] and that PI3K activation can leads to TRPV2 translocation to the plasma membrane [12]. So, we next examined if the presence of TRPV2 at the plasma membrane is dependent on AM and on the integrity of PI3K signaling pathway. To do so, T24/83 cells were treated with either AM (200 nM for 45 min) or AM plus PI3K inhibitor LY294.002 (10 µM, added 5 min prior to AM), and plasma membrane localization was assayed by biotinylation. As shown in figure 3B, AM increased TRPV2 presence at the membrane, and this effect could be inhibited by LY294.002, suggesting the involvement of PI3K signaling in AM effect on TRPV2.

We therefore studied by transwell assay whether LY294.002 could impair PC-3 and T24/83 cell migration. We shown that LY294.002 (10 µM) prevented the stimulatory effect of AM on both PC-3 and T24/83 cells while it did not modified the basal migration of these cells (Fig. 3C). Furthermore AM-induced migration in the presence of siTRPV2 was not affected by the LY294.002 application both in PC-3 and T24/83 cells supporting the specificity of the PIK3 involvement in the AM effect on TRPV2 channel.

## Discussion

AM stimulating activity on tumor progression has been shown on numerous cancers, including prostate but not on bladder carcinoma. To date, two possible mechanisms by which AM supports tumor growth have been postulated. The first possibility is that AM promotes tumor growth by stimulating angiogenesis [5], [6]. The second possible mechanism is that AM directly promotes tumor proliferation and survival. A recent report demonstrated that AM antagonist inhibited the proliferation and invasion of pancreatic cancer cells expressing AM receptors in vitro [20]; however no effect has ever been described on prostate cancer cell migration/invasion and none at all on bladder carcinoma.

In this study we report for the first time that the UC cell line T24/83 also express an AM receptor (CLR/RAMP3) and that AM can increase not only the PCa cell line PC-3 but also the UC cell line T24/83 invasive phenotype in the same way, by stimulating cell adhesion and migration through β1-integrin and FAK activation respectively.

Calcium is an important factor in the process of migration [7]; studies from our laboratory show that the Transient Receptor Potential channel TRPV2 is expressed in the metastatic PCa cell lines, including PC-3 cells, and trpv2 transcript levels are 12 times higher in patients with metastatic cancer (stage M1) compared with primary solid tumors (stages T2a and T2b). Basal activity of this channel, as well as induced, promotes PCa cell migration and invasive phenotype [12], [13]. Furthermore, TRPV2 expression is also increased in higher bladder cancer stage [14]. We demonstrate here that silencing of this channel also drastically reduces the migration and invasion of the UC cell line T24/83. But the most important finding is that TRPV2 modulates AM stimulation of the cell motility and invasion, as shown by the absence of AM effect on adhesion, migration and invasion when TRPV2 expression is knocked down. AM, by acting on TRPV2 translocation to plasma membrane, increased the resting calcium level of both cell lines, which contributed to its effect on PC-3 and T24/83 cell migration.

These data suggest that AM and TRPV2 correlated expression may contribute to creating ideal conditions for metastatic spread in patients with prostate or bladder cancer and could constitute a prospective prognostic marker and a potential therapeutic target to increase the life expectancy of the patients.
